# Isolated lip dermatitis (atopic cheilitis), successfully treated with topical tacrolimus 0.03%

**DOI:** 10.4317/medoral.24230

**Published:** 2020-12-19

**Authors:** Eleni Georgakopoulou, Panagiota Loumou, Aikaterini Grigoraki, Antonios Panagiotopoulos

**Affiliations:** 1 MD, DDS, MSc, PhD. Post Doc Researcher, Department of Histology and Embryology Athens School of Medicine, NKUA, Athens, Greece; 2 MD, DDS, PhD. Second Department of dermatology, Hospital Attikon, Athens, Greece; 3 MD, PhD, Pathologist. Athens School of Medicine, NKUA. Athens, Greece; 4 MD, Dermatologist, Department of Cryosurgery, Hospital Andreas Syggros, Athens Greece

## Abstract

**Background:**

Exfoliative and erosive cheilitis, may be a source of speech and chewing discomfort, but may also be an aesthetic issue for the patients affected. Such a clinical presentation may implicate a variety of inflammatory conditions, including atopic (eczematous) cheilitis. Topical and systemic agents, e.g. corticosteroids, have been used to treat inflammatory lip conditions. Topical tacrolimus has also been used in some inflammatory lip conditions.

**Material and Methods:**

We performed a retrospective clinical analysis of atopic cheilitis patients.

**Results:**

Between 2015 and 2020, we addressed 7 (seven) patients with atopic dermatitis affecting only lips and were diagnosed as atopic-eczematous cheilitis. They were treated with 0.03 per cent topical tacrolimus ointment and responded completely.

**Conclusions:**

These cases represent an underreported atopy / eczema event;-few cases of atopic cheilitis without concomitant dermal lesions appear in the literature. We are also showing and discussing yet another application of tacrolimus in a local atopic form of inflammation affecting the lips.

** Key words:**Atopy, cheilitis, tacrolimus.

## Introduction

Lip is a unique type of tissue sharing features of both skin and oral mucosa. Lips act as a "barrier" for the mouth and receive an abundance of external irritations (1). Lips are also an area of high aesthetic interest (1). A wide variety of local and systemic diseases may present as painful exfoliative lips with swelling and erythema (2). This paper describes 7 cases of patients with sore, erythematous, erosive and swollen lips which were diagnosed as atopic dermatitis isolated to the lips (atopic cheilitis), and were successfully treated with topical tacrolimus 0.03% ointment.

## Material and Methods

This is a retrospective (case series) study and clinical analysis of patients presenting with isolated erosive cheilitis, diagnosed and treated as lip manifestation of atopic dermatitis. All the patients were treated at specialized private oral medicine practices.

## Results

Between 2015 and 2020, 7 patients (4 women and 3 men) presented with the complaint of persistent sore lip lesions. All the female patients were in the 5th decade of their life. Male patients were all over the age of 65. All the patients were referred to Oral medicine specialist by Dermatologists. They had received treatment by dermatologists for “lip dermatitis” consisting of hydrating topical agents, and anti-inflammatory agents. They all had previous history of atopic or eczematous dermatitis.

- Clinical features

**Figure 1:**
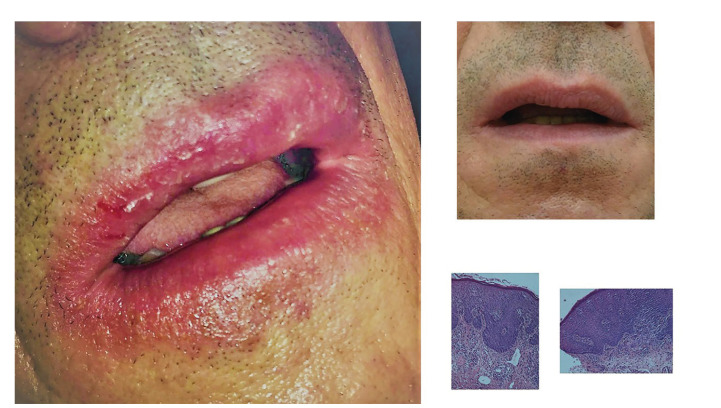
Case1, clinical images before and after treatment and histological features, apparent eosinophilia, edema and hyperemia in the papillary corium.

**Figure 2:**
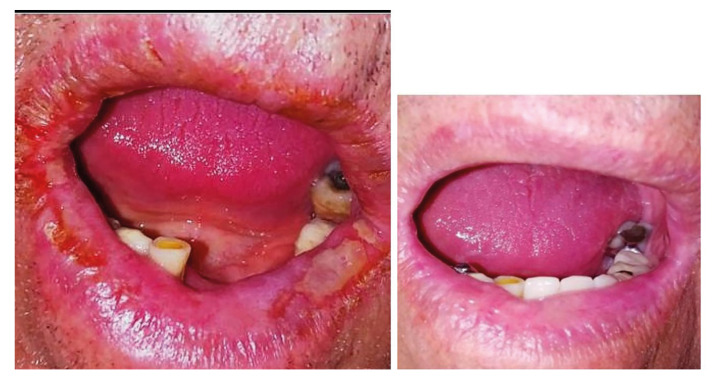
Case 2, clinical images before and after treatment. (notice that the patient did not wear his partial denture in the first visit, as he was not able to eat properly and removing the dentures for cleaning was a painful conditions).

The clinical examination revealed in all the patients dry, painful and exfoliating lips. More severe cases had also swelling of one or both lips, erosions and erythema as well as vertical lip fissures with bleeding tendency (Fig. 1, Fig. 2). None of the patients had other intraoral (eg oral ulcers, dental abscess), dermal or ocular lesions. Neck examination did not reveal tender neck nodes. On palpation the lips were felt firm and tender.

And all the patients complaint of lip pain when opening their mouth for eating and speaking.

- Work up and Response to treatment.

The differential diagnosis based on history and clinical features included atopic cheilitis, contact allergy and actinic cheilitis, while in two patients granulomatous cheilitis was also considered (one of them in Fig. 1). All the patients had previously visited a dermatologist, they all had a history of previous inflammatory dermal disease and all the referring dermatologists appreciated that the lip condition was probably “lip dermatitis”, as part of atopic skin tendency. They had used topical agents (6 patients corticosteroids and 1 pimecrolimus) which did not resolve their lip lesions. The patients were referred to oral medicine specialist for a second opinion regarding diagnosis and treatment.

Most cases were diagnosed according to history and clinical findings. One patient consented to a lip biopsy from the border of the lip vermilion and the mucosa. In this case it was necessary to exclude cheilitis granulomatosa as he had profound and persistent lip swelling (Fig. 1). The histology findings were consistent with atopic cheilitis (Fig. 1).

All patients were treated with topical tacrolimus ointment 0.03% twice daily for 2 weeks with the instruction to avoid sun exposure (especially immediately after the application of the ointment), the application was then tapered to once daily for another 15 days. They were instructed to use small amount of the ointment and to apply it on the lip vermilion and perioral skin when affected. The majority of the patients (5/7) used commercially available tacrolimus 0.03 % ointment, and two used a galenic preparation of tacrolimus 0.03% in oral gel (FAGRON HELLAS). All the patients recovered a normal lip appearance after the treatment (indicative clinical images (Fig. 1, Fig. 2). Response to treatment was also used as a diagnostic criterion in favour of the atopic nature of the lesions. Patients’ characteristics are summarized in Table 1.

**Table 1:**
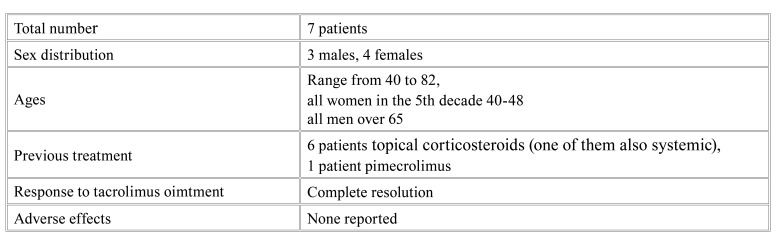
Summary of patients’ characteristics.

## Discussion

Cheilitis (described as non-specific lip inflammation, presenting with desquamation and furrowed lips) is among the minor features of atopic dermatitis (3). Atopic cheilitis is the cheilitis arising secondarily to atopic dermatitis and is also referred as lip dermatitis and eczematous cheilitis. Isolated atopic cheilitis (without dermal lesions) is rather uncommon and it was difficult to identify well documented cases; a large case series of patients being submitted to patch test for investigation of lip dermatitis was diagnosed with atopic diathesis by the North American Contact Dermatitis Group [2001- 2004] (4). Also, an endogenous etiology was the most common cause of cheilitis, in a series of 202 patients with eczematous cheilitis from Singapore (5). Although atopic dermatitis is most prevalent in children, persistent cases affecting adults and adult onset disease is recorded to affect a very small percentage of patients (6). In these patients the head and neck areas are often involved (6). Of note adult disease is more common in women of the fifth decade and men over 65 (6), exactly as the age distribution in our group.

The differential diagnosis includes exfoliative cheilitis, contact cheilitis and actinic cheilitis. Exfoliative cheilitis is lip inflammation, with constant desquamation, usually present in just one lip, especially lower. This form is common among young people with picking and licking lip habits and is associated with stress (7). Persistent lip erosions may also be a feature of actinic cheilitis, and should be included in the differential diagnosis in elderly patients with history of sun exposure (1). Actinic cheilitis is a precancerous lesion, which is typically located on the lower lip and results from excessive and chronic exposure to ultraviolet radiation, if suspected a biopsy is necessary (1). Contact cheilitis shares similar clinical features with atopic cheilitis and it is very difficult to differentiate contact from atopic cheilitis, even with extensive patch testing (8). It would be worth pointing that in such cases we should evaluate both patch testing and response to treatment; for example in the patient of Fig. 1 an extensive patch test was performed and despite testing positive in some substances the lips did not improve when they were withdrawn. Also, he did not respond to treatment with topical corticosteroids. The final diagnosis was based on the histology and response to treatment with tacrolimus (second line treatment for atopic dermatitis). Due to lip swelling (Fig. 1), in this patient we also wanted to exclude granulomatous diseases that cause lip swelling (1). Maybe not in all cases of suspected atopic cheilitis such extensive tests are required, but when it comes to very severe cases it would be worth pursuing to document diagnosis.

The use of topical tacrolimus is indicated for atopic dermatitis resistant to cosrticosteroids (9,10). Tacrolimus (a calcineurin inhibitor) inhibits transduction signaling pathways in T cells after binding to a specific cytoplasmic immunophiline (FKBP12), in the presence of calcium, thus inhibiting transcription and synthesis of IL-2, IL-2, IL-2 5 and other cytokines such as GM-CSF, TNF-α and IFN (10). It was also observed that tacrolimus prevents the release of inflammatory mediators from cutaneous mast cells, basophiles, and eosinophils (10). The tacrolimus cream with 0.1% concentration is indicated for the treatment of moderate to serious atopic dermatitis in adults who did not respond or are intolerant to traditional therapies (9,10). The 0.03% cream is used for the treatment of mild to extreme dermatitis in children (2 years of age and older) who have not responded satisfactorily to conventional therapies (9,10). Of note, another topical calcineurin inhibitor pimecrolimus does not seem to have the same effectiveness in adults with atopic dermatitis (6).

Based on previous reports of use in exfoliative cheilitis we prescribed tacrolimus 0.03% to all the patients as the lip is less keratinized than the skin (11). The patients were instructed to use a small amount of the ointment and were also advised to avoid sun exposure immediately after application. They were also informed about the risk of possible melanotic lesions as adverse effect (12) and we also discussed about the case of oral squamous cell carcinoma in a patient treated with topical tacrolimus for oral lichen planus1 (13), although we explained that mucosal contact is minimal when applying ointment to the lip vermilion. The patients were very compliant and the treatment was uneventful. Unfortunately, with the exception of patients in Fig. 1 and Fig. 2 we do not have long term follow up, these two patients almost two years since the initial episode did not present new lip lesions.

## Conclusions

 In cases of atopic/eczematous cheilitis resistant to corticosteroids or with such severe erosive lesions, the alternative of topical tacrolimus ointment, could be considered, with low concentration (0.03%) being possibly more suiTable choice for the lips. Assessments of medical records, effective communication with dermatologists, which obviously requires basic knowledge and oral medicine expert's experience in dermatology, are important for treating lip lesions; in complex cases in particular.
